# Coordinated Multidisciplinary Care Enables Safe Vaginal Delivery in Complete Heart Block: A Case Report

**DOI:** 10.7759/cureus.89746

**Published:** 2025-08-10

**Authors:** Maria Sonia Rodriguez, Christopher Kaleb Romero Ríos, Catherine S Moreno Cabrera, Lorenzo E Aragón Conrado

**Affiliations:** 1 Obstetrics and Gynaecology, Hospital Militar Escuela "Dr. Alejandro Dávila Bolaños", Managua, NIC; 2 School of Medicine, Hospital Militar Escuela "Dr. Alejandro Dávila Bolaños", Managua, NIC; 3 Medical Education, Hospital Militar Escuela "Dr. Alejandro Dávila Bolaños", Managua, NIC

**Keywords:** congenital atrioventricular block, high-risk obstetrics, multidisciplinary care, pacemaker in pregnancy, pregnancy management

## Abstract

Congenital atrioventricular block (CAVB) is a rare condition that presents unique challenges in pregnancy, especially in patients with permanent pacemaker implantation. Management requires careful coordination to ensure maternal and fetal safety. We report the case of a 35-year-old primigravida with isolated CAVB and a dual-chamber pacemaker (DDD mode), implanted for complete heart block. Pre-pregnancy cardiac evaluation revealed stable ventricular pacing with normal left ventricular function. A multidisciplinary team, including maternal-fetal medicine, cardiology, and electrophysiology, oversaw prenatal care. No changes in pacemaker settings were required during pregnancy or delivery. Labor was electively induced at 39 weeks due to a favorable cervical status and to ensure the availability of specialized staff. Continuous electronic fetal monitoring was employed during labor. Vaginal delivery was achieved without complications, resulting in the birth of a healthy neonate. No maternal or neonatal adverse events occurred in the peripartum period. This case illustrates that with coordinated interdisciplinary care and individualized labor planning, pregnancy and vaginal delivery can be safely achieved in women with CAVB and permanent pacemakers.

## Introduction

The management of cardiovascular diseases during pregnancy poses significant clinical challenges, particularly in rare conditions such as congenital atrioventricular block (CAVB). It is a distinct entity from acquired AV block, as it typically results from fetal developmental abnormalities of the conduction system or maternal autoimmune exposure in utero, rather than from structural heart disease or medication toxicity seen in adults. This disorder, characterized by impaired electrical conduction between the atria and ventricles, can lead to serious maternal and fetal complications, including bradycardia, heart failure, and intrauterine growth restriction [1]. CAVB is an uncommon condition, with an estimated prevalence of approximately 1 in 20,000 live births, and is often associated with maternal autoimmune antibodies, particularly anti-SSA/Ro and anti-SSB/La, which cross the placenta and affect the fetal conduction system [2,3].

The presence of a pacemaker in a pregnant woman adds an additional layer of complexity to clinical management. While this device is essential to prevent symptomatic bradycardia and other cardiovascular complications, it may interact with the physiological changes of pregnancy and require specific adjustments [4]. Follow-up care for these patients should include continuous monitoring and a multidisciplinary team to address potential risks such as arrhythmias, infections, and hemodynamic instability. There is also a need for coordinated planning regarding labor analgesia and delivery mode to mitigate arrhythmic or hemodynamic complications; however, the presence of CAVB and a pacemaker does not necessarily mandate cesarean delivery, as vaginal birth remains a safe option in many cases when appropriately managed [5]. This case report demonstrates the feasibility of vaginal delivery in a woman with CAVB and a pacemaker, emphasizing the importance of multidisciplinary care in achieving favorable maternal and neonatal outcomes.

## Case presentation

A 35-year-old primigravida with CAVB, diagnosed at age 28, received her first pacemaker implantation at the age of 30. Due to disease progression and decreased device efficacy, a new pacemaker was implanted at age 33. Her pre-pregnancy BMI was 27.1, and she reported allergies to ranitidine and pineapple.

**Figure 1:**
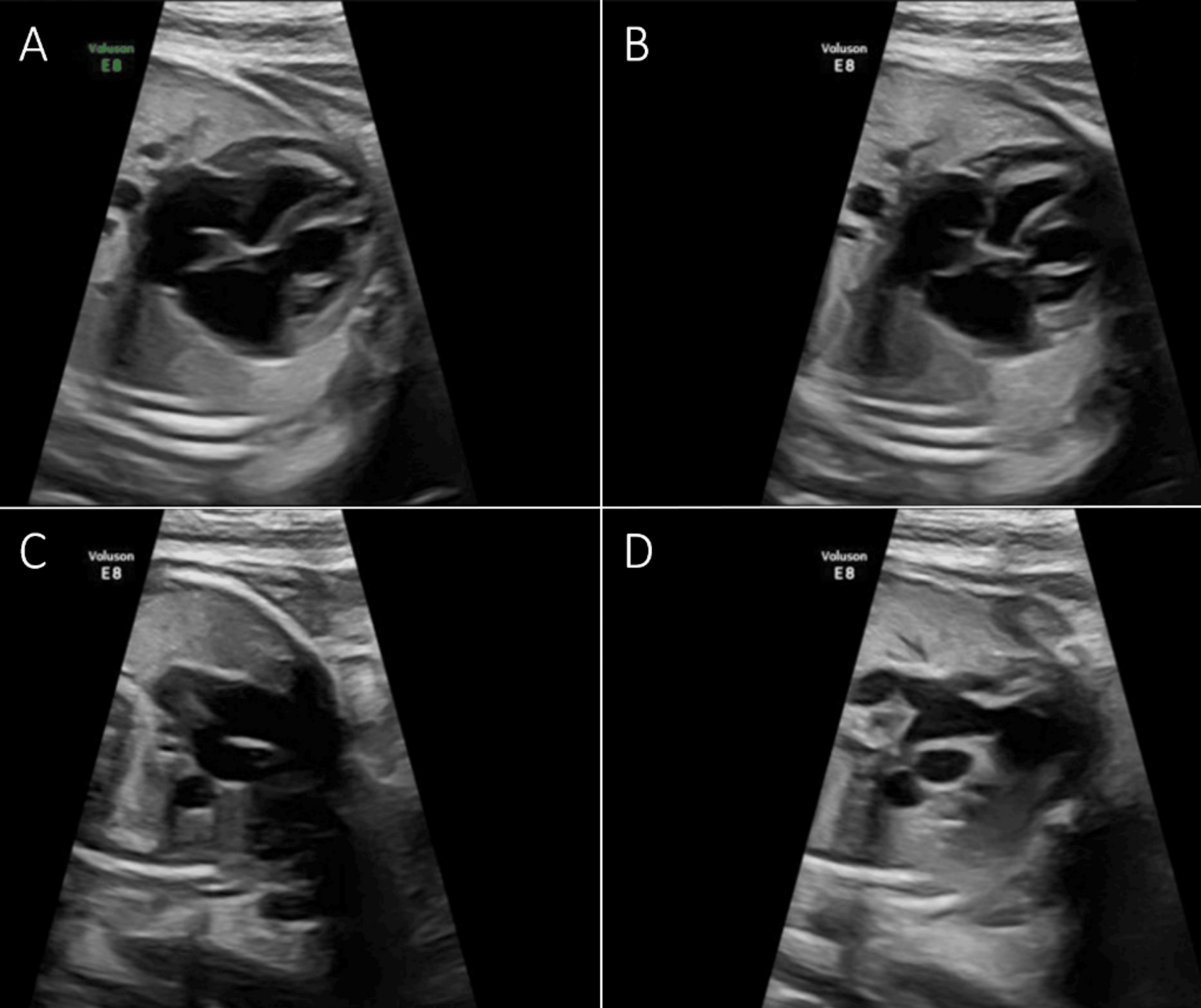
Fetal echocardiography performed at 34 weeks of gestation Fetal echocardiography was performed, demonstrating normal cardiac anatomy and function throughout the cardiac cycle. Images A and C correspond to diastole, showing ventricular relaxation with open atrioventricular valves (A: four-chamber view; C: left ventricular outflow tract). Images B and D correspond to systole, with ventricular contraction and closed atrioventricular valves (B), and aortic valve opening with forward flow through the LVOT (D). No structural abnormalities or functional impairment were identified.

Given her medical history, a multidisciplinary team including Maternal-Fetal Medicine, Electrophysiology, Adult Cardiology, and Pediatric Cardiology managed her care. A fetal echocardiogram at 34 weeks showed a structurally normal heart with a fetal heart rate of 128-136 bpm, patent foramen ovale, and patent ductus arteriosus, without pericardial effusion. Left ventricular systolic and diastolic function was preserved, indicating adequate cardiovascular development for gestational age (Figure 1A-1D). The team agreed to plan delivery at 39 weeks following obstetric indications and guidelines from NYHA and WHO. In cases requiring cesarean section, cardiology was to program the pacemaker to AAI mode before surgery to optimize monitoring and minimize complications. Being a tertiary care center, certified personnel were available for this programming.

The patient remained asymptomatic throughout pregnancy. Labor induction was scheduled at 39 weeks to optimize fetal maturity while minimizing the potential maternal and fetal risks associated with prolonged pregnancy in women with congenital complete atrioventricular block. Given her stable condition and the absence of obstetric contraindications, the goal was to allow for planned delivery under controlled circumstances. 

At her eighth prenatal visit, she presented to the emergency department with normal vital signs and a gravid uterus measuring 33 cm. The fetus was in cephalic presentation with a heart rate of 145 bpm. No uterine contractions or cervical changes were noted; a reactive non-stress test was normal

Laboratory tests and obstetric ultrasound showed a grade III anterior placenta, amniotic fluid index of 8.85 cm, fetal heart rate of 137 bpm, and fetal biometric measurements consistent with a 3160 g fetus at the 26th growth percentile.

The patient was admitted to the high-risk obstetrics unit for vaginal labor induction with misoprostol 25 micrograms vaginally starting at 07:00 hours. Vital signs remained stable. Cardiac rhythm was intermittently assessed throughout labor via clinical evaluation and ECG; continuous external fetal and uterine monitoring was performed using cardiotocography. No maternal bradycardia, arrhythmias, or pacemaker-related issues were observed. By 10:00 hours, she reported labor pain and clear vaginal fluid discharge, with uterine contractions of moderate intensity occurring twice every 10 minutes. Fetal movements were active, and fetal heart rate ranged between 132 and 140 bpm. Vaginal exam revealed a closed, thick, posterior cervix, and premature rupture of membranes was diagnosed.

Two hours later, with stable vital signs, she was transferred to the labor room with a Bishop score of 7, cervical dilation of 5 cm, 70% effacement, and regular contractions. Labor progressed spontaneously with the administration of epidural anesthesia.

At 17:21 hours, the cervix was fully dilated, and complete effacement was achieved. The second stage of labor lasted approximately 30 minutes. A right lateral episiotomy was performed without complications. A healthy female neonate was delivered at an estimated gestational age of 38 weeks (by Capurro test), with Apgar scores of 8 and 9 at 1 and 5 minutes, respectively, and a birth weight of 2940 g. Active management of the third stage included 10 units of intramuscular oxytocin, uterine massage, and controlled cord traction. The placenta and membranes were delivered intact, with approximately 250 ml of blood loss. No cervical or vaginal lacerations were found.

Postpartum, the patient and newborn were transferred to the recovery room. Hematological control after 24 hours showed no abnormalities. Given the satisfactory clinical course and absence of immediate complications, a follow-up appointment was scheduled for seven days post-discharge, during which no adverse events related to delivery were noted.

## Discussion

Complete CAVB is a rare condition, with an incidence of approximately 1 in 15,000 to 20,000 live births [6]. It occurs more frequently in females, with studies reporting that about 58% of affected individuals are female [4]. The pathogenesis is often associated with maternal autoantibodies, specifically anti-Ro/SSA and anti-La/SSB, which can cross the placenta and damage the fetal atrioventricular node, leading to cardiac conduction block [4,6].

In the present case, the patient was diagnosed during childhood with isolated CAVB. There was no history of maternal autoimmune disease. Although anti-Ro/SSA and anti-La/SSB testing was indicated during pregnancy to evaluate potential autoimmune etiology, the test could not be performed due to unavailability of reagents in the country at the time. The clinical presentation and the absence of maternal autoimmune symptoms favored a non-autoimmune, isolated form of CAVB.

Management of CAVB typically involves pacemaker implantation, especially in symptomatic patients or those with significant bradycardia, left ventricular dysfunction, wide QRS complexes, or prolonged QT interval [3,6]. It has been reported that up to 77% of patients with CAVB require pacemaker implantation, with a mean age at implantation of 1.9 years [7].

During pregnancy, women with pacemakers require close multidisciplinary monitoring to ensure optimal device function and maternal hemodynamic stability. Although pregnancy-induced hormonal and hemodynamic changes can potentially affect pacemaker thresholds or sensing parameters, no alterations were observed in this case. Device interrogation performed each trimester showed stable parameters, with no requirement for reprogramming. Available literature suggests that women with preexisting pacemakers generally have a low risk of complications during pregnancy and delivery [8,9]. In this case, the patient underwent regular monitoring each trimester by Electrophysiology and Cardiology services, following international guidelines.

Mode of delivery in women with CAVB does not necessarily require cesarean section. Several studies have demonstrated that, in the absence of specific obstetric or cardiovascular indications, vaginal delivery can be safe and appropriate [10,11]. Hidaka et al. and Sundararaman et al. concluded that women with permanent pacemakers implanted prior to conception can be safely managed with vaginal delivery under adequate surveillance [8,12].

However, cesarean section may be indicated in certain circumstances, such as severe maternal bradycardia, hemodynamic instability during labor, or the need for temporary pacemaker implantation before delivery [11-13]. Additionally, standard obstetric indications such as fetal distress, malpresentation, or failure to progress in labor also warrant cesarean delivery.

From the fetal perspective, the primary complication of CAVB is the development of cardiac conduction block, ranging from first to third degree. Exposure to maternal anti-Ro/SSA and anti-La/SSB antibodies can cause inflammation and fibrosis of the fetal atrioventricular node, resulting in bradycardia, hydrops fetalis, or even intrauterine fetal demise [7]. Mortality associated with CAVB is significant, with reported rates up to 20% [14,15].

Prenatal diagnosis relies mainly on fetal echocardiography and Doppler ultrasound, which assess fetal heart rhythm and detect atrioventricular conduction abnormalities. These modalities are particularly important in high-risk pregnancies, such as those with maternal anti-Ro/SSA and anti-La/SSB antibodies. Surveillance typically begins around 16 weeks’ gestation, as conduction block can progress rapidly. Doppler ultrasound enables measurement of the atrioventricular interval, aiding in early detection of conduction delays [16].

In this case, the neonate was born at term, with no signs of bradycardia or structural anomalies. Postnatal evaluation included physical examination, ECG, and echocardiography by the pediatric cardiology team, all of which were normal. No evidence of congenital conduction defects was found.

The patient was counseled regarding the importance of postpartum follow-up with Cardiology and the pacemaker clinic. A device check was scheduled six weeks postpartum, and routine long-term surveillance was recommended. Breastfeeding was not contraindicated, as no medication or condition limited lactation in this patient, and her cardiac status remained stable.

Neonates with CAVB may require pacemaker implantation in up to 64% of cases within the first year of life. Growth retardation has been documented in infants with second- or third-degree atrioventricular block, with no significant catch-up growth during childhood. These infants may also have associated complex congenital heart defects, further worsening the prognosis [17].

The diagnostic and management limitations encountered in resource-constrained settings. Although fetal echocardiography was available and utilized for prenatal assessment, maternal anti-Ro/SSA and anti-La/SSB antibody testing could not be performed due to the unavailability of reagents at the national level, preventing confirmation of an autoimmune etiology. Despite this limitation, the patient exhibited no clinical signs of connective tissue disease, and serial fetal assessments, including echocardiography and biophysical profiles, remained reassuring throughout pregnancy. Given the patient's stable cardiac status, normal pacemaker function, and absence of obstetric contraindications, the decision to proceed with labor induction at 39 weeks and plan for vaginal delivery was made following multidisciplinary evaluation [8-12].

**Figure 2:**
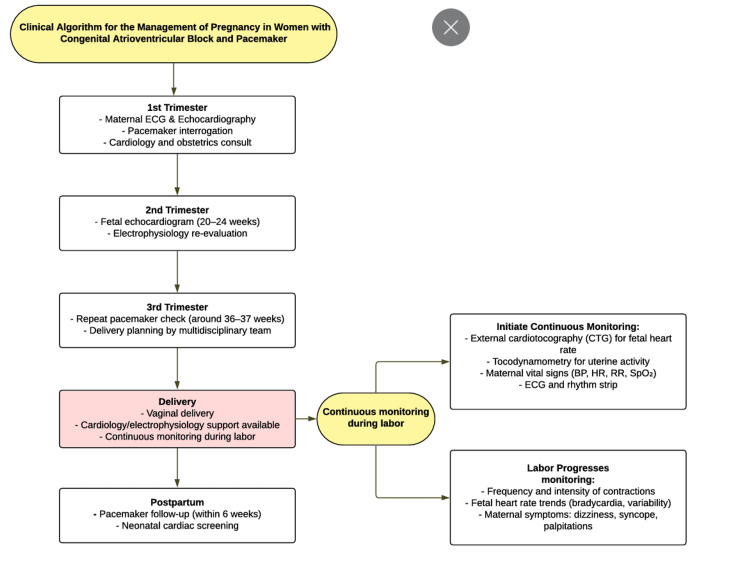
Clinical Management Algorithm This flowchart summarizes the multidisciplinary care pathway we followed for managing pregnant women with congenital complete atrioventricular block and permanent pacemakers. It outlines the recommended monitoring schedule, timing of evaluations, and key considerations for ensuring safe vaginal delivery and postpartum care.

Given the complexity and potential risks associated with managing pregnant women with congenital complete atrioventricular block, our multidisciplinary team followed a standardized clinical algorithm to guide monitoring frequency, specialist consultations, and delivery planning. This structured approach allowed for the timely identification of maternal and fetal complications and facilitated coordinated care (Figure 2).

## Conclusions

Managing pregnant patients with CAVB and implanted pacemakers presents distinct clinical challenges that require careful, individualized planning and a coordinated multidisciplinary approach. This case demonstrates that, despite the complexity of the condition, favorable maternal and neonatal outcomes can be achieved through comprehensive monitoring and close collaboration among obstetricians, maternal-fetal medicine specialists, cardiologists, and pediatricians. Notably, it underscores that vaginal delivery is a safe and often preferred option when adequate resources and expertise are available, challenging the notion that cesarean section is routinely required. Optimal management involves anticipating and addressing potential complications such as arrhythmias and hemodynamic changes throughout pregnancy and delivery. Postpartum care, including maternal cardiology follow-up and neonatal surveillance by pediatric cardiology, completes the continuum of care. Ultimately, this case highlights the importance of proactive, integrated strategies that prioritize maternal cardiac stability and fetal well-being, while emphasizing the need for standardized protocols to guide clinicians in managing high-risk pregnancies complicated by congenital cardiac conduction disorders and pacemaker dependency.
